# A Modified Cooperative A* Algorithm for the Simultaneous Motion of Multiple Microparts on a “Smart Platform” with Electrostatic Fields

**DOI:** 10.3390/mi9110548

**Published:** 2018-10-25

**Authors:** Georgia Kritikou, Nikos Lamprianidis, Nikos Aspragathos

**Affiliations:** Robotics Group, Department of Mechanical Engineering and Aeronautics, University of Patras, 265 04 Rio, Greece; nlamprianidis@upnet.gr (N.L.); asprag@mech.upatras.gr (N.A.)

**Keywords:** microparts manipulation, smart platform, electrostatic phenomena, activation algorithms, Configuration-Space (C-Space), static and moving obstacles, modified A* algorithm

## Abstract

In this article, a method for microparts parallel manipulation with electrostatic forces, applied by conductive electrodes embedded on a Programmable “Smart Platform”, is introduced. The design of the platform and the layout of the electrodes underneath the rectangular microparts with respect to the platform’s geometry are presented. The electrostatic phenomena that result to the electrostatic forces applied to the microparts by the activated electrodes of the “Smart Platform” are studied in detail. Algorithms for the activation of the platform’s electrodes for the motion of the rectangular microparts are introduced and their motion is simulated. The Configuration-Space (C-Space) of the microparts on the “Smart Platform” is defined taking into account the static obstacles that are placed on the platform and the rest of moving microparts. Considering the layout of the platform, the activation algorithms, the motion and the C-Space of the microparts, a modified A* algorithm is proposed and the best path for every moving rectangular micropart on the “Smart Platform”, is computed with respect to time. Simulated experiments are presented to demonstrate the effectiveness of the proposed approach and the results are discussed.

## 1. Introduction

The last decades, the micro-electro-mechanical system (MEMS) industry requests new manipulation methods for the mass parallel sorting and assembly of MEMS’ micro-objects. For the time being, serial methods are used in the synthesis of complex MEMS, reclaiming pipets [1], piezoelectric actuators, [2] grippers [3] and microrobots [4,5]. However, the serial manipulation methods for the handling of micro-objects, are time consuming, that is undesirable for the mass production of Microelectromechanical Systems.

Contactless Micro-Manipulation is a promising, evolving field that may contribute in the most effective production of MEMS, since parallel (simultaneous) manipulation of multiple microparts could be achieved. Methods for manipulation could be divided in sensorless and sensor based for the localization of the microparts. Regards to the sensorless manipulation of microparts, Kavraki and Boheringer [6,7] introduced the concept of programmable vector force fields, which move microparts to equilibrium positions, where they should be assembled. Programmable 2-D force fields that “trap” the microparts in the desired locations were proposed by Lazarou et al. [8] and Xidias [9], while the micromanipulation with 3-D force fields for microparts sorting and assembly applications was presented in Reference [10]. However, programmable fields was just the beginning, while the last two decades applications for microparts handling with sensors feedback for the localization using forces whose magnitude is considerable in micro dimensions, have been introduced in the relevant publications. 

Pneumatic forces, caused by the activation of air-flow MEMS surfaces, have contributed to the planar motion of microparts with various shapes and under high speed [11,12,13], however the achieved positioning accuracy of the microparts is not satisfactory. The traverse of microparts with high speed has also been succeeded on an air/liquid surface, by the aid of magnetic fields [14], as well as the motion of paramagnetic micro-spheres by using magnetic fields was simulated [15]. Berkelman and Dzadovsky succeeded to partially rotate and levitate micro-objects with the aid of an array of micro-coils [16].

The contactless handling of microparts with or without sensors using Electrostatic Fields is a research area with great interest. Karl Boehringer [17] was the first who tried to trap microparts on vibrating plates with the aid of attractive electrostatic forces. A different vibrating plate where mili- and micro-objects were located with electrostatic fields was introduced in Reference [18]. The authors in Reference [19] proposed a device for the positioning of mili-polymer parts with electrostatic forces, while a technique for the handling of glass panels for the manufacturing of liquid crystal display devices was presented in Reference [20].

A Programmable Platform for the micropart’s motion using electrostatic forces was proposed by Lazarou et al. [21,22]. The Programmable Platform consists by an electrodes array embedded on a plastic substrate covered by a dielectric membrane, where microparts with electrodes underneath are manipulated by the activations of the platform electrodes with a constant potential. The activation methods for the vertical, horizontal and diagonal motion were described in detail. The feasible motions of a single micropart were simulated in Matlab/Simulink (2011a) and the measurements of the position and the velocity with respect to time were presented. 

The optimal path computation on a discretized 2D workspace is a common problem in robotics and has attracted the attention of a lot of researchers. In the following the main and more recent publications for multi-robot motion planning on graphs are presented. A very effective search algorithm for the shortest path computation is A* algorithm [23], which is considered as an advanced version of the best first search algorithm [24], reclaiming both the advantages of uniform costs and greedy searches using a fitness function [24]. In order to decrease the computation time, which was the weakness of A* algorithm, Warren [25] modified the classic A* algorithm by implementing a loose search on a fine grid. Since then a lot of modified versions of A* algorithm were introduced as Basic Theta [25] and D* [26]. 

Taking into account the priority of the robots during their simultaneous motion, for the path planning of the robots either decoupled or coupled methods are considered [23]. Modified A* algorithms have been used in robotics for the path planning of single or multiple mobile robots [26,27]. In [28] the authors proposed an on-line, decoupled method for the parallel motion of mobile robots in a continuous workspace. The D* algorithm was used for the independent path computation of each robot and the collision avoidance between them was achieved by delaying the next steps of the robots with lower priority. A decoupled method was proposed in Reference [29], where the A* algorithm was used for the path computation based on a heuristic function, where both the time and the collision avoidance with the static obstacles were considered. The priority of the robots was decided by genetic algorithms and a Multi Neuron Heuristic Search was used for the optimization of the results after the path search. 

The M* is another modified A* algorithm that computes the optimum path of multiple robots considering the collision avoidance between them [30]. The M* algorithm is not a fixed-path method [23], since at every step it optimizes the paths of the microparts considering the collision avoidance between them. The method that is presented by the authors attempts to combine both the coupled and decoupled methods. The CA* (Cooperative A*) [31] algorithm, computes the unique path of every robot in time-space. The CA* is a decoupled path computation method that considers the paths of the robots with higher priority and does not permit to the rest of the simultaneously moving robots to follow the same route in time-space. 

The main difference between the robots and microparts manipulation on the Programmable Platform is the activation mode. The robots move by the action of their motors, while the microparts should be moved by the activation of the suitable platform electrodes. Therefore, path planning algorithms widely used in robotics may be adapted for the path planning of microparts considering the activation difference. Specifically, when the robots are displaced on a graph from their current node to the next, they can change their velocity or even stop on the graph edge so to reach their goal with a time-delay [28,29]. On the other hand, the moving microparts make discrete motions from their current node to the next in constant time-steps, due to the platform activation method, which does not permit them to change their velocity.

Sarantoglou et al. [32] computed the paths of two simultaneously moving microparts, due to the activations of an array of *N* × *N* rectangular conductive electrodes, using a prioritized planning method [23]. The Dijkstra algorithm [23] was used as the route finder algorithm on a coordination space, however the complexity of this algorithm is quite high. The off-line path planning of microparts with modified A* algorithm is a research area with very limited number of publications. One attempt was presented by Chowdury et al. [33], where the D* lite [34] algorithm was used in order to compute the path of microrobots, which were displaced on a discrete area, due to magnetic fields induced by micro-coils. 

Considering the previous works, the simultaneous decoupled manipulation of multiple moving microparts with electrostatic fields so to be adapted in batch sorting and assembly applications is an open research area with limited publications. An extended study of the Programmable Platform (the “Smart Platform”—SP) introduced in Reference [22], for the parallel motion of microparts is presented. The Configuration Space and the State Space of the microparts considering the activation modes of the SP electrodes for the microparts motion is defined. In this paper, the micro-Cooperative A* algorithm (μCA*) is introduced for the decoupled path determination of the rectangular microparts in a discretized time-space. The μCA* adapts the modes of the activation and the constraints that are introduced, due to the microparts motions and considers all the configurations that result in the collision with the static obstacles and the rest of moving microparts. In the frame of μCA* algorithm a new heuristic function is proposed, which takes into account the number of the displacements that the microparts have to make in order to reach their goal and the distance from the rest moving microparts. The heuristic function contributes in the optimization of the path computation, as it makes the μCA* algorithm to search for feasible paths in regions, which are not simultaneously occupied by multiple microparts. 

This article is structured as it follows. In Section 2 the Hierarchy of the method for parallel manipulation on the “Smart Platform” (SP) with electrostatic fields is presented, in Section 3 the Finite Elements Method (F.E.M). determinations of the electrostatic interaction between the Smart Platform electrodes and the microparts are included; and in Section 4 the Configuration Space and the Activation Space of the microparts are described. In Section 5 the State Space of the microparts is specified; and in Section 6 the μCA* algorithm and the new heuristic function is explained in detail. Finally, in Section 7 the computations of μCA* algorithm reclaiming the new heuristic function and the heuristic function of the A* algorithm are discussed.

## 2. The Proposed Approach for Parallel Manipulation of Microparts

**Figure 1:**
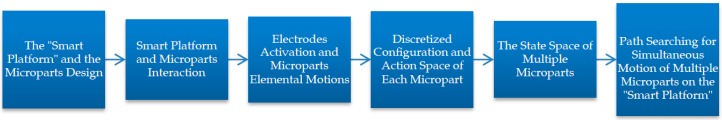
The proposed approach for simultaneous manipulation of multiple microparts step-by-step.

In this work, a full study for the simultaneous manipulation of multiple microparts on the “Smart Platform” is presented. In Figure 1, the proposed method for the parallel motion of multiple rectangular plastic microparts is illustrated step by step. An alternative layout of the microparts electrodes, considering authors’ previous publications [22], is proposed, towards decreasing the mass of the microparts. Based on the new layout suitable activation algorithms for the microparts horizontal and vertical elemental motion are introduced. Rectangular microparts whose dimensions vary are simulated with F.E.M. analysis and the electrostatic and friction forces that are applied on them are computed.

The “Smart Platform” is considered as a discretized area with static obstacles. Thus, the State-Space of the Platform is described considering the C-Space and the Action Space of the microparts. The configurations that result in static obstacles—microparts and microparts—microparts collision are studied, and the constraints for the collision avoidance between them are introduced, taking into account the constraints that are imposed by the activation mode. Considering the proposed constraints the obstacles State Space and the free State-Space in the “Smart Platform” are determined. 

The μCA* algorithm and a new heuristic function are presented in detail. The path of multiple microparts is computed with the μCA* algorithm using the new heuristic function and the simulated results are selected and compared with the μCA* simulated computations that are implemented using the conventional distance function of A* algorithm. 

## 3. The Smart Platform and the Rectangular Microparts

**Figure 2:**
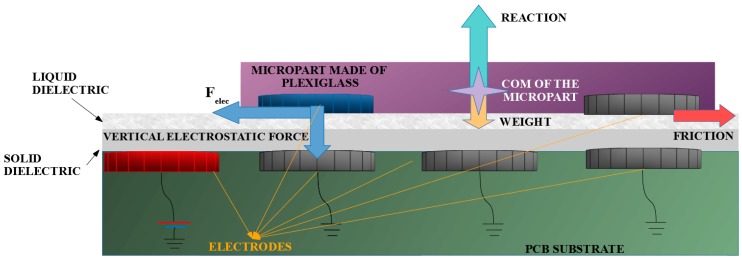
Shortcut of the “Smart Platform”.

In this Section, the “Smart Platform” (SP) and the rectangular microparts are presented. The SP is a PCB substrate, where *N* × *N* grounded circular conductive electrodes are embedded and two dielectric layers are placed on top of the substrate. The microparts made of plexiglass, with circular conductive electrodes embedded in their bottom, can be manipulated on the upper surface of the platform, by the activation of the suitable platform electrodes. A liquid dielectric is selected as the upper layer of the “Smart Platform”, in order to reduce the friction forces presented during the microparts motion. It is supposed that the location of the microparts on the platform is known using a vision sensor system. The design of the platform was introduced by authors of this paper in References [21,22,35], and a shortcut of the “Smart Platform” is illustrated in Figure 2.

**Figure 3:**
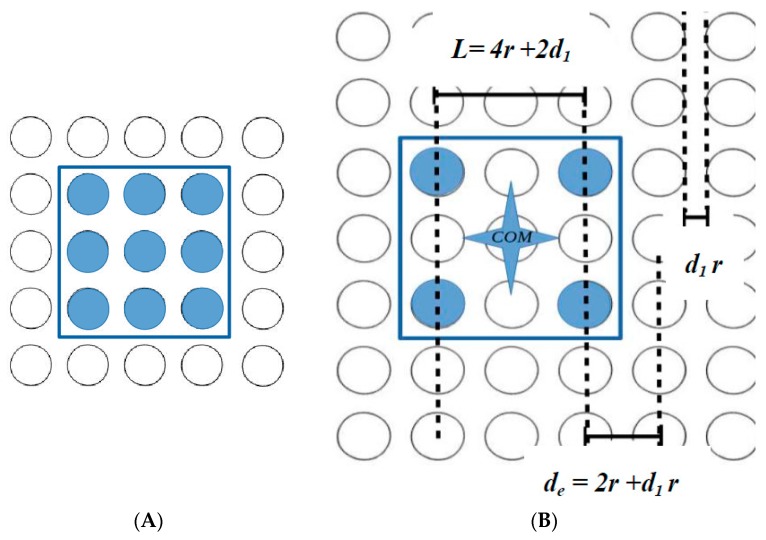
(**A**) The layout of a rectangular micropart embedded with 3 × 3 circular conductive electrodes [22]. (**B**) The layout of a rectangular micropart embedded with 2 × 2 circular conductive electrodes.

The layout of 3 × 3 electrodes (blue circles Figure 3A) underneath of the rectangular microparts, was designed with respect to the geometry of the platform by Lazarou et al. [21,22]. In the present work, regarding to the decrease of the size and the mass of the microparts, an alternative layout of the electrodes of the microparts is proposed in this work; 2 × 2 circular electrodes (blue circles), whose radius *r* is equal to the radius of the platform electrodes, are placed underneath of the micropart, as it is shown in Figure 3B. The distance between the centers of two neighbor platform electrodes is equal to $\;d_{e}=2r+d_{1}r$, where $\;d_{1}\;\in \left[0.5,\;0.896\right]\;$ while the distance between two neighbor micropart electrodes is equal to $\;L=4r+2d_{1}r$. The product $d_{1}r$ is the distance between the edges of two neighbor electrodes considering the limits for arc avoidance [21]. Since the micropart moves by the platform electrodes activation (up/down and left/right), then the equilibrium location is that one, where two non-diagonal of the micropart electrodes coincide with the activated ones of the “Smart Platform”. Therefore, its center of mass (COM) coincides with the center of a platform electrode, as it is shown in Figure 3.

### 3.1. F.E.M. Analysis of The Electric Field 

In previous publications, [21,35], the interaction between a couple of parallel oppositely charged electrodes with two dielectric layers between them was considered. The electrostatic forces between the activated platform electrodes and the neighbor micropart’s electrode with respect to the electrical and geometrical parameters of the platform were computed. The function of the electrostatic force that resulted from these computations was used for the simulation of the motion of a single micropart in References [21,35]. Authors of this work determined that for the successive handling of the microparts on arrays with conductive electrodes and dielectric layers, the radius of the electrodes has to vary from 15 μm to 100 μm. 

In this work, the interactions between the activated electrodes of a region of the “Smart Platform” and the electrodes of a rectangular micropart made of plexiglass are studied with F.E.M. analysis. The target is to visualize the electrostatic fields that are presented on the platform and to explain in detail the physics of the microparts motion. Moreover, the magnitudes of the electrostatic forces, which make the microparts to leave their static condition are determined.

#### 3.1.1. Studying the Charging of the Micropart Electrodes with F.E.M. Analysis

**Figure 4:**
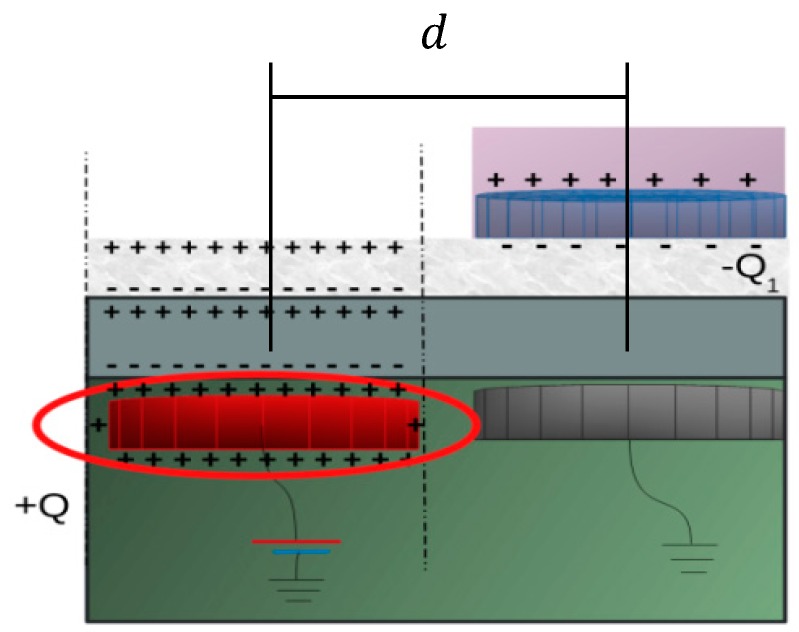
Charge induction due to the polarization of the dielectrics.

The manipulation of a micropart is based on the successive charging of the micropart electrodes by induction, after the activation of the neighbor platform electrodes. An example of the method is illustrated in Figure 4, where after the charging of the platform electrode with +Q the dielectrics of the SP get polarized and finally the negative charge of the uncharged micropart electrode is induced at its bottom side. In [21,35] it was considered that the micropart electrode succeeds to induce charge so that $\;\left|+Q\right|=\left|-Q_{1}\right|$, which is reconsidered by computing the induced charge of the micropart electrodes. 

A 6 × 8 electrodes platform was simulated in with electrodes whose radius r varies among 15 μm to 100 μm. The length of the micropart $L_{m}$ that is placed on the top of the SP is equal to $\;L_{m}=6r+3d_{1}r$, its volume is equal to $\;L_{m}\times \;L_{m}\times \frac{r}{2}$ and the $\;d_{1}$ constant is equal to 0.7. The liquid Al_2_O_3_ is used as the dielectric lubricant at the upper surface of the platform, where its static friction coefficient is equal to $\mu_{s}=0.3\;\;$ [36] and its electric permittivity is equal to $\varepsilon_{r}=8$. The micropart is made of plexiglass ($\rho_{plexiglass}=1.18\cdot 10^{6}\;\mu g/{\mu m}^{3}$). 

**Figure 5:**
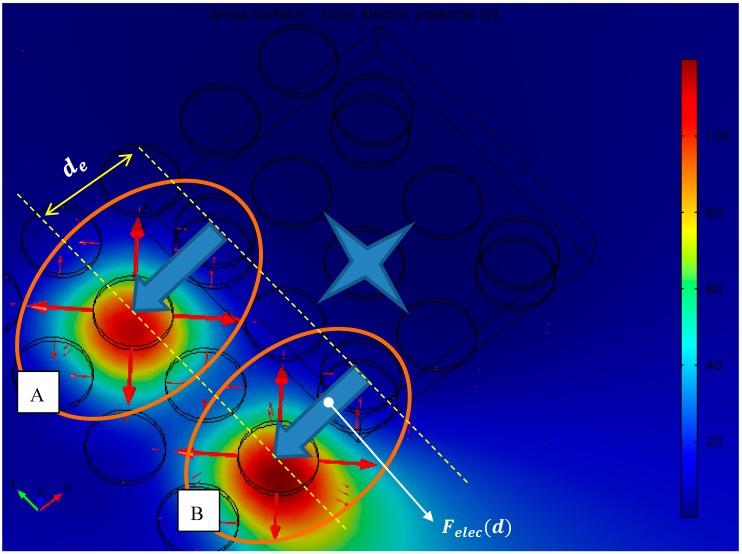
Snapshot of the simulated “Smart Platform” isolating the interactions between the platform and micropart electrodes.

A snapshot of the platform when a micropart equilibrates on its top is illustrated in Figure 5, in the case that r=25 μm. As it is shown in Figure 5, the COM of the micropart coincides with the center of a SP electrode. Taking into account the geometry of the micropart at least two platform electrodes should be activated so not to be overturned. The value of +150 V is applied to the platform electrodes enclosed in A and B ellipses. The selected potential is chosen considering the computations that were implemented for electrodes with the same geometry and dimensions, presented by authors of this work in Reference [35]. The horizontal distance between the SP and the micropart electrodes of A and B ellipses of Figure 5 is equal to $\;d_{e}$. The red vectors of Figure 5 represent the electrostatic fields, which surround the SP electrodes. The interactions of the electrodes enclosed in ellipse A of Figure 5 are isolated so to be studied the charging of the micropart electrode.

**Figure 6:**
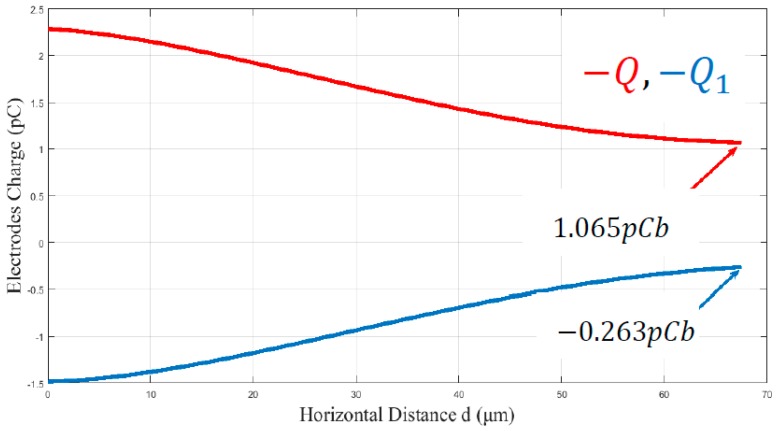
Comparing the Charge of platform and Micropart electrodes with respect to their centers distance—Platform Electrode—Micropart Electrode.

In the graph of Figure 6, the Finite Elements Method (F.E.M.) determinations of the charge versus the horizontal distance between the couple of electrodes of ellipse A (Figure 5) are shown. When the distance between the centers of platform-micropart electrodes is equal to $d_{e}$ the platform electrode is charged (+Q=+1.065 pCb), due to the positive potential. A negative charge ($-Q_{1}=\;-0.263\;\mathrm{pCb}$) is induced at the bottom side of the micropart electrode, but $\;\left|+Q\right|\neq \left|-Q_{1}\right|$. As the distance  d gets decreased the attractive forces between the electrodes and the fringe fields are more intense [37], so their charge gets increased. From the computations of Figure 6, it results that in any distance  d, $\left|+Q\right|=\left|-Q_{1}\right|$.

#### 3.1.2. The Electrostatic Forces that Are Applied to the Microparts 

For the successive manipulation of the microparts, it has to be known the potential, which can make the micropart to leave its current position, considering its dimensions. As it is shown in Figure 2, after the charging of the SP electrodes, the micropart electrode is attracted horizontally by the corresponding activated platform electrode. 

The total electrostatic force $\;F_{elec}^{\;s}\;\left(d\right)$ that is applied to the COM of the micropart during its motion from its current to its next equilibrium position is the sum of the isolated interactions ($F_{elec}\left(d\right)$) between the SP and the neighbor micropart electrodes as the one that is illustrated in Figure 5 in Ellipse A. Specifically, the force $\;F_{elec}^{\;s}\;\left(d\right)$ applied to the COM of the micropart is given by:

(1) $$\;\;\;F_{elec}^{s}\;\left(d\right)=\;\overset{\begin{array}{c} number\;of\\ activated\;electrodes \end{array}}{\underset{s=1}{\sum }}\;F_{elec}\left(d\right)\;.\;$$

The $F_{elec}^{2}\;\left(d\right)$ corresponds to the electrostatic force applied to the COM for the minimum number of activations, which can move the micropart. The target is to find such a potential so that the weight of the micropart not to be considerable and the electrostatic force $F_{elec}^{2}\;\left(d_{e}=2r+d_{1}r\right)$ to overcome the static friction force that is applied to the micropart. Different potentials are applied to two of the platform electrodes (Figure 5) and the electrostatic force $F_{elec}^{2}\;\left(d_{e}\;\right)$ was measured with F.E.M. analysis, while the static friction force was computed considering the weight and the vertical electrostatic force that is applied to the microparts. In Table 1 the maximum potentials for which the $F_{elec}^{2}\;\left(d_{e}\right)$ electrostatic force overcame the static friction force, for varying dimensions of the microparts, are included. 

**Figure 7:**
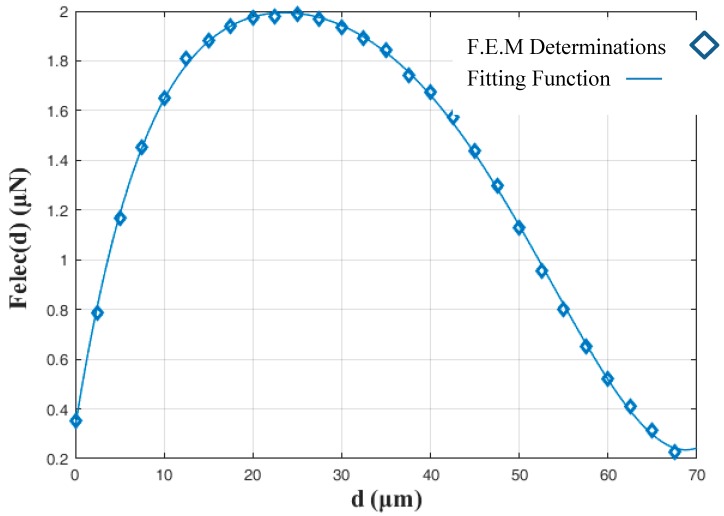
Horizontal Electrostatic Force F.E.M. determinations ($F_{elec}\left(d\right)$) with respect to the distance between the centers of platform—micropart electrodes.

For the dynamic model of the micropart the $F_{elec}\left(d\right)$ function is requested. Thus, the force $F_{elec}\left(d\right)$ (Figure 5—Ellipse A) is computed with respect to d for the different dimensions of the microparts. In Figure 7 the F.E.M. determinations of $F_{elec}\left(d\right)$ versus d are illustrated for the case that r=25 μm. As it is shown, the $F_{elec}\left(d\right)$ is maximum when d=25 μm, while the micropart has not reached yet its goal configuration and its electrodes have not totally overlapped the corresponding platform electrodes. As it was discussed in detail in Reference [35], the $F_{elec}\left(d\right)$ depends on the fringe fields that are more intense when the electrodes are partially covered. The determinations are fitted with a 6th grade polynomial function, which is used in the following for the micropart motion simulation. 

### 3.2. Motion of the Micropart under the Electrostatic Force

In this Section the dynamic model of the moving micropart is studied. The differential equation that describes the motion of a micropart from its current position to the next is:

(2) $$\;m\;\frac{du}{dt}+b\cdot u=F_{elec}^{\;2}\;\left(d\right),\;$$

where m is the mass of the microparts, b is the damping coefficient whose value is considered with respect to the mass, the dimensions of the micropart and the dynamic viscosity $\eta_{l}=5{\;\mu g\;\mu m}^{-1}s^{-1}$ of the liquid dielectric layer $\left(b=\frac{\eta_{l}\cdot L^{2}}{m_{total}\cdot \frac{r}{2}}\right)$ [22], and u  is the velocity of the micropart. 

**Figure 8:**
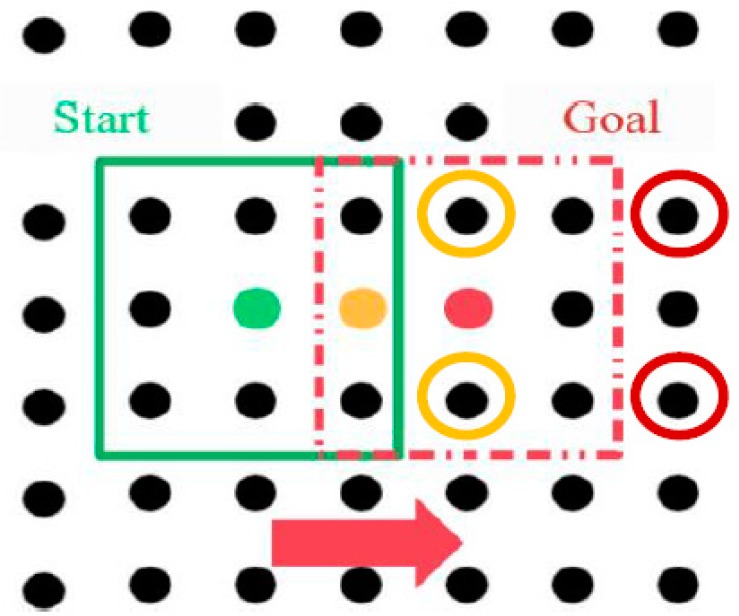
Motion of the micropart that is simulated in Matlab/Simulink.

**Figure 9:**
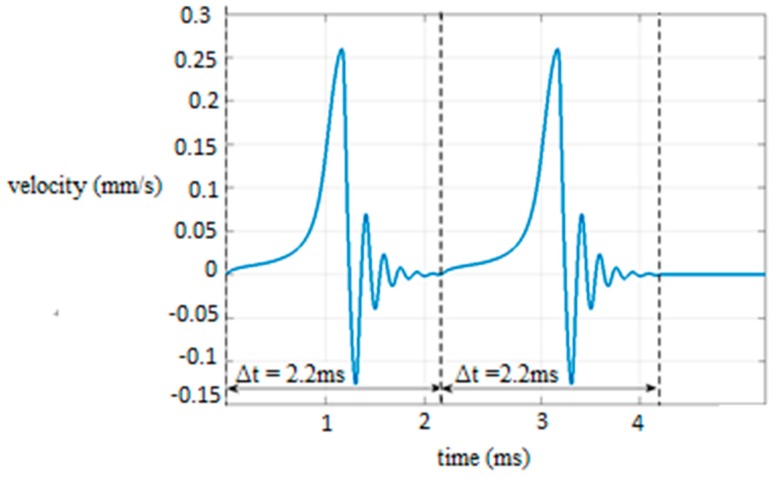
Graph of the velocity of the micropart with respect to time for the displacement of the COM of the micropart after two activations of the “Smart Platform”.

The displacement of Figure 8 is simulated in Matlab/Simulink, and the micropart’s velocity results with respect to time for the case that $r=35\;\mu m\;\mathrm{and}\;V^{+}=100\;V$ are illustrated in Figure 9. The micropart oscillates from 1  to 2.2 ms when it completes its first displacement so that its COM to coincide with the center of the yellow platform electrode and be static (u=0). The same motion is implemented for the micropart’s displacement from the yellow to the red platform electrode (Figure 8) where the SP electrodes in the red circles were activated and the electrodes in the yellow circles are deactivated simultaneously. So, every step of motion of the micropart lasts a constant time Δt  and the variation of the velocity vs. time is identical in each step. 

## 4. The Configuration and the Action Space of the Microparts

**Figure 10:**
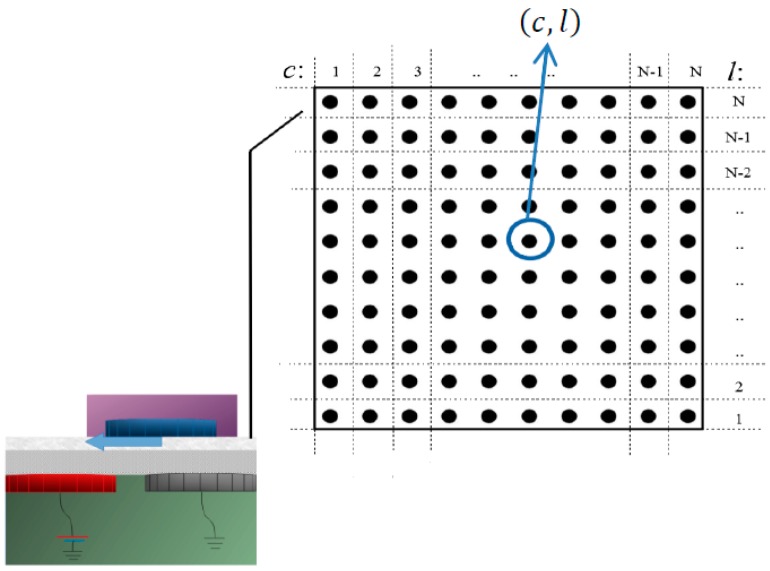
Representation of the electrodes array of the “Smart Platform”.

In this section the Configuration and the Action Space of the microparts is described. The mobility of the microparts on the “Smart Platform” can be represented as a discrete motion planning problem on the “Smart Platform”, which is a finite region where the distance $d_{e\;}$ between the centers of neighboring electrodes is constant. As it is described in Section 3.2, the displacement of the micropart from its current position to the next lasts a constant time  Δt. During a time step the micropart either moves to the next equilibrium position or remains stationary. Thus, the electrodes array of the SP can be represented by a grid whose nodes and edges represent the centers of the electrodes and the connecting lines between them respectively, as it is shown in Figure 10. 

When a micropart equilibrates on the SP, its COM coincides with a SP electrode  (c, l), where c  is the number of the column, while l  is the number of the line. The coordinates of the electrode underneath the COM of the micropart represents the current position of the micropart. So the current configuration of the micropart is equal to $\;q^{i\;}=\left(c^{i},l^{i}\right)\;$ where  i∈[1,k],  where k  is the total number of microparts that are moving simultaneously on the SP. Each distinct equilibrium location of the micropart is a configuration $\;q^{i\;}=\left(c^{i},l^{i}\right),\;$ since the rotation is not permitted and the set of all feasible configurations of the micropart consist its discretized Configuration Space $C^{i}\;$[23]. 

In this work, the COM of the micropart is going to move on the electrodes right/left down/up from its current position. So, a 2-geometry neighborhood problem is studied [38], where the distance from the current position of the COM to each one of the four possible transitions is equal to $\;d_{e\;}$ and the time step  Δt. Let the Configuration Space (C)  to be the set of all (c, l),  electrodes and the action space to be equal to:

(3) $$\;U=\left\{v_{1},v_{2},v_{3},v_{4},v_{5}\right\}=\left\{\left(1,0\right),\left(-1,0\right),\left(0,-1\right),\left(0,1\right),\;\left(0,0\right)\right\}=U\left(q^{i}\right),\;\forall q^{i}\in C^{i}.\;$$

The transition equation is given by Reference [23]:

(4) $$U(q^{i}{}^{\prime })\;=f\left(q^{i}\right)=q^{i}+v_{a},\;\;\;v_{a},\in U.$$

**Figure 11:**
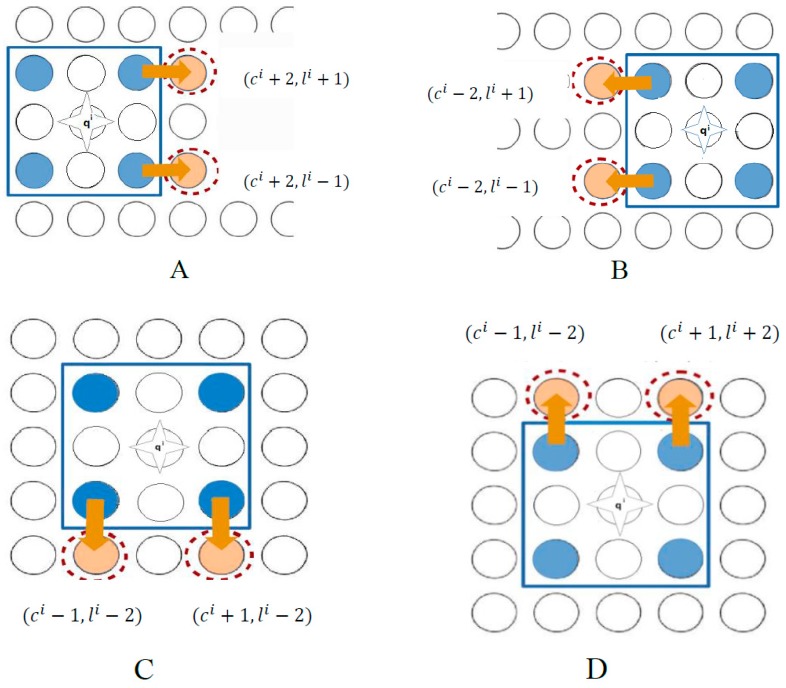
Electrodes activation algorithms for (**A**) right (**B**) left (**C**) down and (**D**) up motion of the microparts on the “Smart Platform”.

In order to change the configuration of the micropart from $q^{i}\;$ to $\;q^{i}{}^{\prime }$, a specific combination of platform electrodes have to be actuated, depending on, which of the five is permitted. In Table 2, the electrodes that are activated for the right/left/up/down/no motion of the microparts are included. The activations for a single displacement of the micropart from its current to its next equilibrium position are illustrated in Figure 11A–D.

### Mapping of the Platform to Graph Considering the $C_{free}$ Space of the Microparts

**Figure 12:**
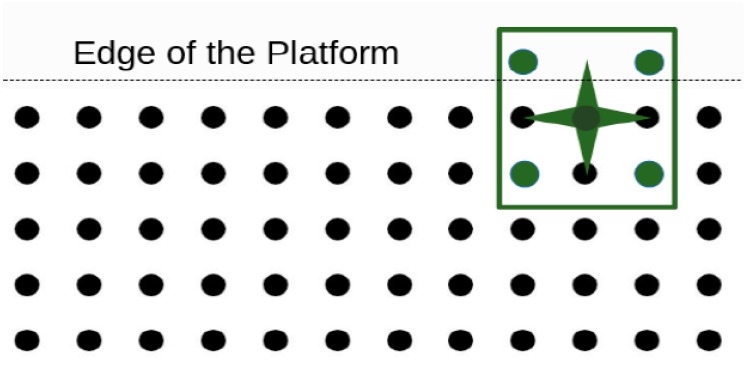
Non-feasible configuration when the electrodes at the edge of the platform are not rejected from the micro-graph.

In this section the $C_{free}$ Space is defined considering the limits of the SP and the Static Obstacles. At the beginning of the computations all the electrodes of the “Smart Platform” are considered as the C-Space (C) of each micropart. Due to the rectangular microparts shape, configurations as the one illustrated in Figure 12, cannot be achieved, since there no SP electrodes whose activations can drive it there. Considering that the $\;C_{SPE\;}$ includes all the electrodes, which correspond to configurations of the microparts at the edges of the “Smart Platform”, the Free Configuration-Space of the microparts is described as:

(5) $$\;C_{free}^{\prime }=\frac{\left(N\;\times \;N\right)}{\;\;\;C_{SPE\;\;}}\;.\;\;$$

**Figure 13:**
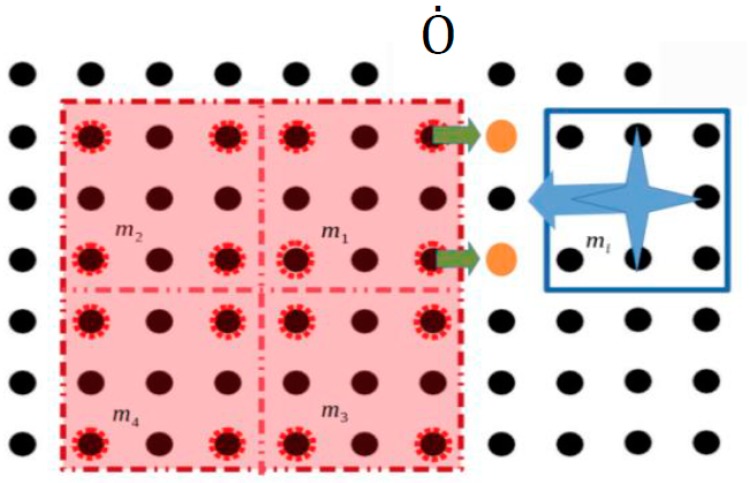
Influence of the activation to the group of assembled microparts.

The visual sensors system of the platform detects all the electrodes that are occupied by the static obstacles belonging to the Ȯ space. Since the static obstacles represent microparts that are in fixed positions on the platform, the activations of platform electrodes which are sufficiently close apply to them electrostatic forces. An example is illustrated in Figure 13 where a group of four assembled microparts is static on the platform and a micropart $m_{i\;}$ is going to move towards them. However, the activation of the platform electrodes (yellow discs) in order to displace $\;m_{i\;}$, apply forces to the neighbor electrodes of the $m_{1\;}$ micropart.

**Figure 14:**
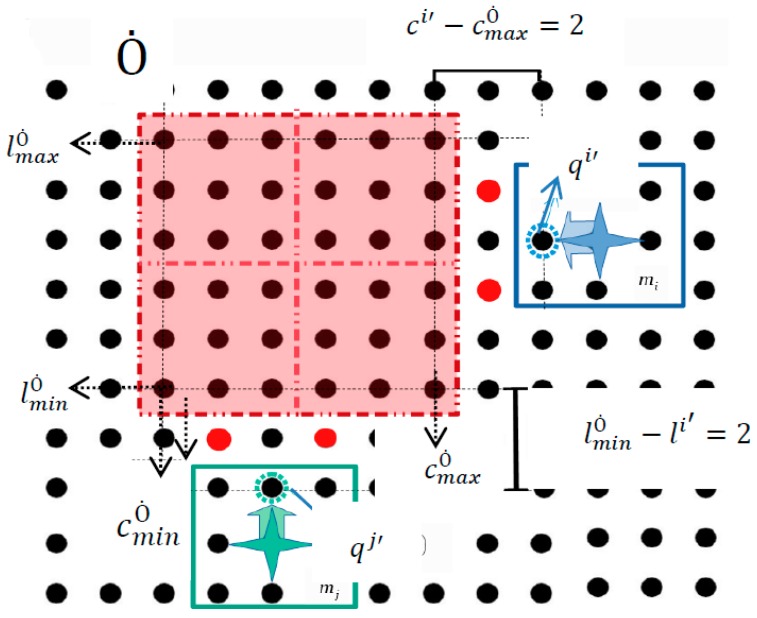
The micropart cannot move to the left as it does not satisfy the static obstacles constraints.

For the computation of the Configuration Space of the static obstacles, the obstacle region Ȯ is considered. Every electrode (c,l) ∈ Ȯ is taken into account as occupied and is described by $\left(c^{Ȯ},l^{Ȯ}\right)$. The current configuration of every micropart $\;m_{i\;}$ where i∈ [1,k] is $\;q^{i}$, while $q^{i}{}^{\prime }$ is the next. In order to be described the constraints for the motion of the microparts around the Static Obstacles the minimum and maximum values of the coordinates of the Ȯ are considered ($c_{min}^{Ȯ},\;c_{max}^{Ȯ},l_{min}^{Ȯ},\;l_{max}^{Ȯ})$. As it is shown in Figure 14:

- If the micropart is going to move horizontally the constraints $\;c_{min}^{Ȯ}-c_{i^{\prime }}>2\;or\;c_{i^{\prime }}-c_{max}^{Ȯ}>2\;$ have to be satisfied. In the example that is illustrated in Figure 14, if the blue micropart selected the configuration $\;q^{i}{}^{\prime }$, the corresponding activations of the SP electrodes would influence the Static Obstacles region and would result in the undesired results of Figure 13.
- If the micropart is going to move vertically the next configuration should satisfy the constraints $\;l_{min}^{Ȯ}-l_{i^{\prime }}>2\;or\;l_{i^{\prime }}-\;l_{max}^{Ȯ}>2$. Similarly with the case of the horizontal motion, in the example of Figure 14 the $\;m_{j\;}$ micropart cannot move up successively because $\;l_{min}^{Ȯ}-l_{i^{\prime }}=\;2$.

Considering the proposed constraints the Configuration Space of the Static Obstacles is equal to:

(6) $$\;C_{obs}=Ȯ\cup \;\left\{\begin{array}{c} \forall \;\left(c,l\right)\;,where\\ \;c_{min}^{Ȯ}-2\leq \;c\leq c_{max}^{Ȯ}+2\;\;and\;\;l_{min}^{Ȯ}-2\;\leq \;l\leq l_{max}^{Ȯ}+2\; \end{array}\right\}.\;$$

**Figure 15:**
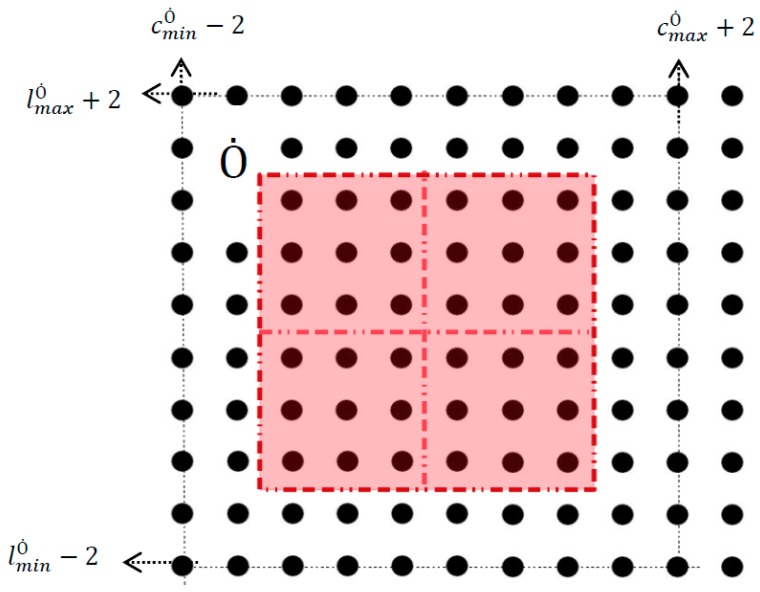
The $C_{obs}$ Space of the “Smart Platform”.

The $C_{obs}$ space is shown in Figure 15. Taking into account both the computation of the $C_{obs}$ and the $C_{free}^{\prime }$ space that was presented in Section 5, the $C_{free}$ Space is described by:

(7) $$\;C_{free}=\;C_{free}^{\prime }/C_{obs}\;.$$

An undirected [39], graph $G\left(M,E\right),\;\left[M=\;C_{free}\;\&\;E\;the\;edges\right]$ is created, covering the free C-Space. The graph has nodes, which correspond to the centers of the circular electrodes of the platform, which are members of the $C_{free},$ and the edges, which are equal to the distance between the centers of the electrodes ($d_{e})$. 

## 5. The State Space of the Microparts on the “Smart Platform”

In order to solve the motion planning problem, for the parallel motion of the microparts on the “Smart Platform” in the time-space, the Free State-Space $X_{free}$ of the microparts has to be specified. The State Space X of the microparts on the “Smart Platform” is equal to  M×T∈ ${\mathbb{N}}^{3}$, where T  is the discretized bounded time [23], which is equal to:

(8) $$\;T=\;\left[t_{0},t_{0}+\Delta t,t_{0}+2\Delta t\ldots ,\;t_{0}+s\Delta t,\;\ldots \;t_{0}+S\Delta t\right]\;,\;$$

where $t_{0}\;$ is the time that all the microparts start to follow their route simultaneously and $t_{0}+S\Delta t\;$ is the time when the last moving microparts complete their motion on the SP. 

The configuration of the $m_{i}$ micropart in time-space is represented by $\;q_{t_{0}+s\Delta t}^{i}=\left(c_{i},l_{i},t_{0}+s\Delta t\right)=\;\left(q^{i},t_{0}+s\Delta t\right)$. The microparts share the same workspace and a path for every micropart is requested on the graph, where the collisions with other microparts should be avoided. 

The $X_{obs}\;$ includes all the configurations that should not be reached simultaneously by the microparts so to avoid the collision between them. The $X_{obs},\;$ whose computation method will be described in this Section will be used for the decoupled path search of every micropart in the Free State Space $\;X_{free}$ that is described by:

(9) $$X_{free}=X/X_{obs}.$$

### Collision Avoidance between the Simultaneously Moving Microparts

For the problem of the collision avoidance during the parallel motion of microparts the pair of $\;m_{i}\;\&\;m_{j}$ microparts, is studied. The target is to compute the subspace of X that includes all the states x that conclude in the collision of $m_{i}$ with $m_{j}$ given by:

(10) $$X_{obs}^{i,j}=\;\left\{m_{i}\left(q_{t_{0}+s\Delta t}^{i}\right)\cap m_{j}\left(q_{t_{0}+s\Delta t}^{j}\right)\neq 0\right\}.$$

**Figure 16:**
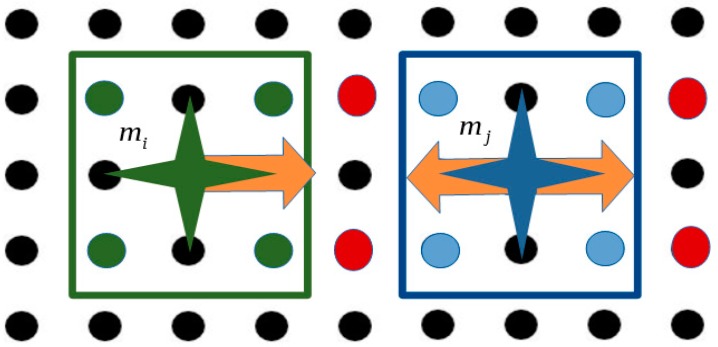
Both the $m_{i}\;\&\;m_{j}$ microparts should be displaced to the right side, but the activation for the $\;m_{i}$ influences the $m_{j}$ micropart and so it cannot implement its motion.

For the computation of $X_{obs}^{ij}$ both the distances between the microparts and the activations of the platform electrodes for their manipulation, are considered. Specifically, in the example of Figure 16 both $\;m_{i}\;\&\;m_{j}$ are going to move to the right side. The horizontal distance between microparts’ COM seems to be sufficient so not to collide. However, as it is shown in Figure 16 the activations of the platform electrodes for the displacement of the $\;m_{i}$ micropart influence the $m_{j}$ micropart. Specifically, because of the activated electrodes (red discs of Figure 16) the $\;F_{elec}^{total}\left(d\right)$ force applied to the $m_{j}$ micropart is equal to zero, so the $m_{j}$ micropart cannot move. Thus, when the $\;m_{i}$ micropart will complete its displacement the microparts will collide undesirably. 

**Figure 17:**
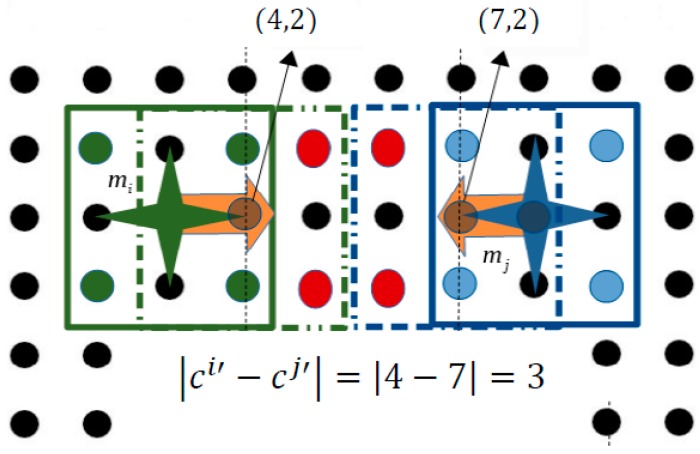
Collision due to wrong simultaneous activations.

In Figure 17 another “wrong” activation on the SP, is illustrated. Specifically, the configurations (4,2) and (7,2) are selected as the next configurations for $\;m_{i}$ and $m_{j}$ micropart respectively. As it is shown, the microparts are not going to collide during their motion and the activations (red discs) for the transition of $m_{i}$ micropart do not influence the motion of $m_{j}$ micropart. However, when both the microparts reach their final configurations (dashed rectangles of Figure 17), the microparts will oscillate and collide.

**Figure 18:**
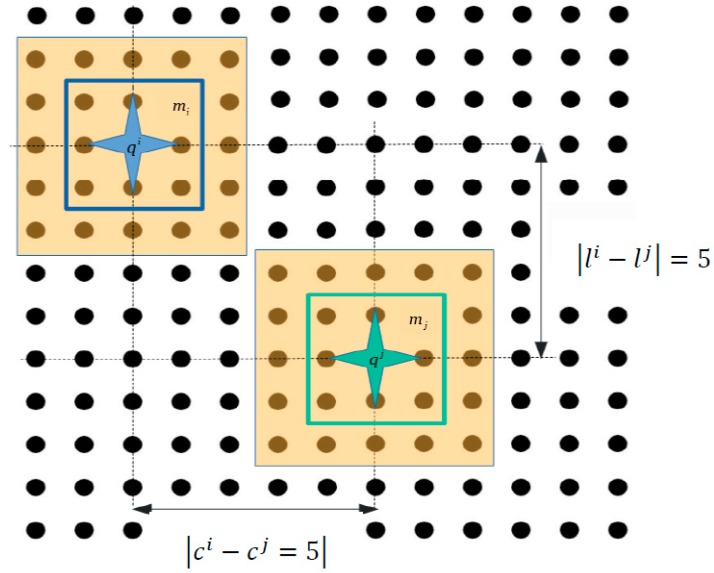
Configurations where the microparts do not collide.

Let’s assume that the configuration of the $\;m_{i}\;$ micropart at is the $q_{t_{0}+\left(s-1\right)\Delta t}^{i}$ and the $\;q_{t_{0}+\left(s-1\right)\Delta t}^{j}$ of the $m_{j}\;$ micropart respectively, while $q_{t_{0}+s\Delta t}^{i}=\left(c^{i},\;l^{i},t_{0}+s\Delta t\right)\;$ and $q_{t_{0}+s\Delta t}^{j}=\left(c^{j},\;l^{j},t_{0}+s\Delta t\right)$ are the next. As it is shown if Figure 18, when the coordinates of the configurations $q_{t_{0}+\left(s-1\right)\Delta t}^{i}\;\mathrm{and}\;q_{t_{0}+\left(s-1\right)\Delta t}^{j}\;$ satisfiy the constraints $\;\left|l^{i}-l^{j}\;\right|\geq 5$ and $\left|c^{i}-c^{j}\;\right|\geq 5,$ the distance between the moving microparts is large enough so not to collide. However, these constraints limit feasible configurations for the $\;m_{j}\;$ micropart so; in the next paragraphs of this Section all the possible scenarios for the most optimum next configurations of the $\;m_{j}$ micropart are studied.

**Figure 19:**
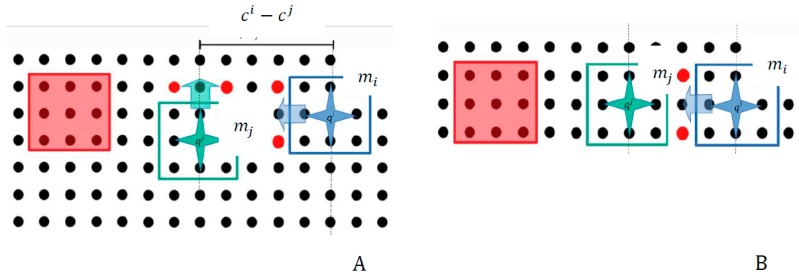
Study of the constraints for the parallel motion of two microparts. (**A**) the distance between the microparts seems sufficient; (**B**) finally the activation for $m_{i}$ influences the motion of $m_{j}$.

**Figure 20:**
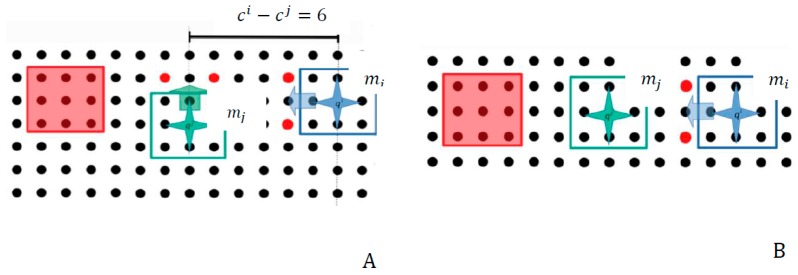
Best Configuration Choice for collision avoidance. (**A**) the sufficient distance between the $m_{i}$ and $m_{j}$; (**B**) the sufficiency of their distance is certified.

Let’s compare two possible cases of two similar scenarios, which are illustrated in Figure 19 and Figure 20, respectively. In Figure 19 the $\;m_{i}$ micropart, which has high priority, has selected to make two discrete motions to the left side of the SP. As it is illustrated in Figure 19 the configurations of the $\;m_{i}$ and $\;m_{j}$ accord with the constraint $c^{i}-c^{j}=5$. At the beginning of their motion it seems that the distance between them is sufficient so to avoid the collision between them. However, when the $\;m_{i}$ makes its second displacement to the left side, the $\;m_{j}$ micropart is influenced by the corresponding SP activations and the microparts finally collide. In the case of Figure 20A the constraint $c^{i}-c^{j}\geq 6\;$ is satisfied by the microparts configurations. As it is illustrated in Figure 20B the $\;m_{j}$ can move without to be influenced by the SP activations, which drive the $\;m_{i}$ to move to the left. 

**Figure 21:**
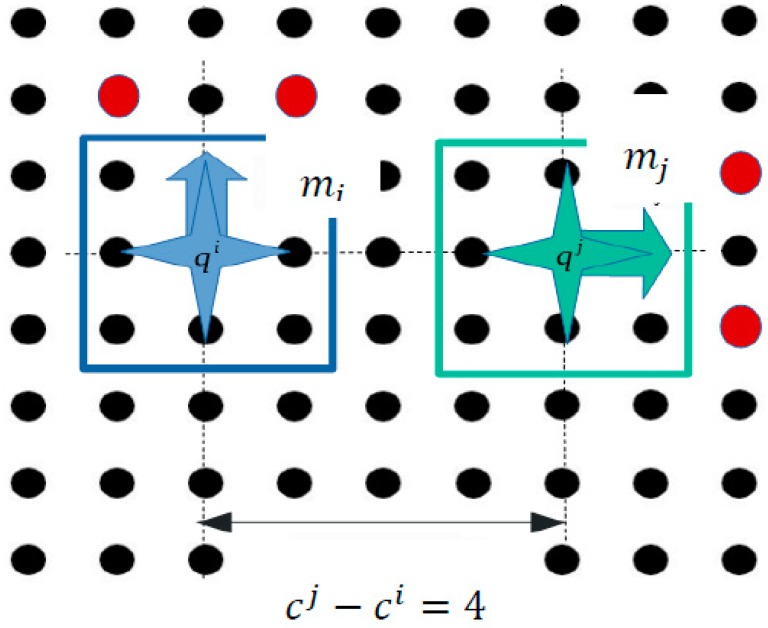
Constraints for the parallel motion of two microparts which diverge the one from the other.

One last case is illustrated in Figure 21, where the $\;m_{i}$ micropart is moving up, while the $\;m_{j}$ is safely moved to the right side of the SP. Considering both the examples shown in Figure 20 and Figure 21, the constraints that should be satisfied are different when the microparts come close and when they diverge the one from other. 

The main topic of this work is the optimum path computation using a decoupled approach. Thus, in Table 3 all the constraints for the computation of $\;q_{t_{0}+s\Delta t}^{j}$ configuration considering the $\;q_{t_{0}+\left(s-1\right)\Delta t}^{i}\;\mathrm{and}\;\;q_{t_{0}+\left(s-1\right)\Delta t}^{j}$ are included. It is taken into account that the $\;m_{i}$ has higher priority than the $\;m_{j}$. Moreover, for the contents of Table 3 it is considered that $c^{i}>c^{j}$ and $\;l^{i}>l^{j}$, and that $\;c^{i}-c^{j}\leq 6\;and\;\;l^{i}-l^{j}\leq 6$.

Τhus, at every time step from $t_{0}+\left(s-1\right)\Delta t$ to $t_{0}+s\Delta t$ the $X_{obs}^{ij}$ is determined considering the constraints of Table 3. Thus, the resulting $X_{obs\;}$ space is described by:

(11) $$\;X_{obs}=\overset{k}{\underset{j\;=\;i+1}{\cup }}X_{obs}^{ij},\;\;\;i\in \left[1,k-1\right]\;$$

So the $X_{free}$ is computed by Equation (9) and the $m_{j}$ micropart will choose its next configuration so that $\;q_{t_{0}+\left(s-1\right)\Delta t}^{j}\in X_{free}.$

## 6. Path Searching for the Parallel Motion of the Rectangular Microparts on the “Smart Platform” 

In Section 4 and Section 5 a brief study of the Configuration and the State Space of the microparts on the “Smart Platform” was presented. In Section 6 all the proposed constraints for the collision avoidance between the simultaneously moving microparts on the graph are considered for the path computation for the parallel motion of multiple microparts. A modified version of the Cooperative A* (CA*) algorithm [31] is proposed for the parallel motion of the microparts on the SP, called micro-CA* (μCA*). A new heuristic function is used for the estimation of the next configuration of the microparts, as well as the incorporation of the microparts constraints in collision avoidance. 

### 6.1. The Heuristic Function of the μCA* Algorithm

As in A* algorithm [23,26], an OPEN list is formulated where the computations of the heuristic function for the feasible configurations of the microparts, are included. The $q_{t_{0}+\left(s-1\right)\Delta t\;}^{i}$ configuration is considered as the current position of the $\;m_{i}$ micropart while $q_{t_{0}+s\Delta t}^{i}$ is the next that is selected among the members of $\;Q^{i^{\prime }}=\left\{\left(q^{i}+v_{a},\;\forall \;v_{a}\;\in U\;,\;t_{0}+s\Delta t\right)\right\}$ space. In the μCA*, the next node estimation and the collision avoidance check are implemented at $t_{0}+\left(s-1\right)\Delta t$. 

Compared to M* algorithm [30] the computed path of the microparts with higher priority does not change during the route search of the microparts with lower priority. Thus, the heuristic function that is used for the computations of μCA* algorithm considers constraints, which estimate the $q_{t_{0}+s\Delta t}^{i\prime }$ configuration. Specifically, it takes into account parameters which “predict” that the next steps that will be chosen by the microparts will drive them in safe positions which will prevent them from being “trapped”. 

The criterion for the selection of $q_{t_{0}+s\Delta t}^{i}$ is the function $f\left(q^{i^{\prime }},q^{w},\;t_{0}+s\Delta t\right),$ which is computed only when the constraints of Table 3 of Section 5 are satisfied $\forall \;q_{t_{0}+s\Delta t}^{i}\;\in \;Q^{i^{\prime }}$:

(12) $$\begin{array}{c} f\left(q^{i^{\prime }},q^{w=1\;:\;i-1},\;t_{0}+s\Delta t\right)\;=g\left(q^{i^{\prime }}\right)+h\left(q^{i^{\prime }},q^{w},\;t_{0}+s\Delta t\right)=\\ \;\left|q^{i,goal}-\;q^{i\prime }\right|+\;\frac{i-1}{\sum_{w=1}^{i-1}\left|q_{t_{0}+s\Delta t}^{i}-q_{t_{0}+s\Delta t}^{w}\right|}.\; \end{array}$$

The $g\left(q^{i^{\prime }}\right)$ function estimates the remaining number of discrete displacements of the $\;m_{i}$ micropart to reach its final configuration $q^{i,goal}$ using the Manhattan metric [23]. The distance cost is a parameter, which is not considerable in this problem—as the edges which connect the nodes of the graph have the identical length $d_{e}$. The motions of the microparts are discrete, thus the number of the movements that the micropart will implement in order to reach its goal, is the same whichever route it follows. The $g\left(q^{i^{\prime }}\right)$ function is used for verifying that the micropart comes closer to its goal configuration, thus its minimum value is requested. 

The function $h\left(q^{i^{\prime }},q^{w},\;t_{0}+s\Delta t\right)$ is the heuristic function which contributes to the minimization of the total time $\;t_{0}+S\Delta t$. For the heuristic the $\overset{i-1}{\underset{w=1}{\sum }}\left|q_{t_{0}+s\Delta t}^{i}-q_{t_{0}+s\Delta t}^{w}\right|$ function is considered, which is the sum of the Manhattan metric of the $q_{t_{0}+s\Delta t}^{i}$ configuration from the rest of the microparts that are moving with higher priority, whose position at $t_{0}+s\Delta t$ is already computed. The $\overset{i-1}{\underset{w=1}{\sum }}\left|q_{t_{0}+s\Delta t}^{i}-q_{t_{0}+s\Delta t}^{w}\right|$ function contributes to the selection of the next configuration that is far from the rest of moving microparts at $t_{0}+s\Delta t,$ which protects it from choosing configurations, where it can be trapped and remain stationary for a long time. Since the $g\left(q^{i^{\prime }}\right)$ function is part of the $f\left(q^{i^{\prime }},q^{w},\;t_{0}+s\Delta t\right)$ function, the criterion for the selection of the optimum $q_{t_{0}+s\Delta t}^{i}$ configuration is the minimum value of the $f\left(q^{i^{\prime }},q^{w},\;t_{0}+s\Delta t\right)$ function, thus the inverse of $\overset{i-1}{\underset{w=1}{\sum }}\left|q_{t_{0}+s\Delta t}^{i}-q_{t_{0}+s\Delta t}^{w}\right|$ multiplied by i−1 is added to $\;g\left(q^{i^{\prime }}\right)$. The $\;\frac{i-1}{\sum_{w=1}^{i-1}\left|q_{t_{0}+s\Delta t}^{i}-q_{t_{0}+s\Delta t}^{w}\right|}$ function is finally considered as the formula of the proposed function. 

**Figure 22:**
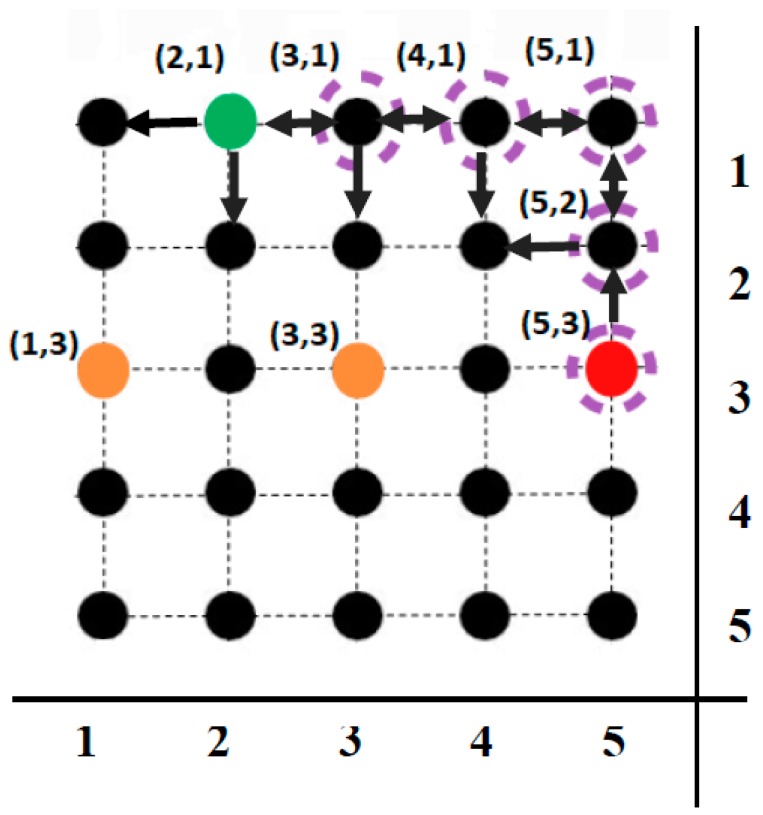
The heuristic function results for collision avoidance of the $A^{3}$ microparts from the static $A^{1},A^{2}$ microparts.

A simple example for the efficiency of $f\left(q^{i^{\prime }},q^{w},\;t_{0}+s\Delta t\right)$ function is presented below. Three point microparts $\left(A^{1},A^{2},A^{3}\right)$ are on a 5 × 5 node graph and it is considered that the $A^{1}\;\&\;A^{2}$ microparts are static in their current configuration. As it is shown in Figure 22 the function is computed for every feasible configuration of the $A^{3}$ micropart and the results are used for its path cost estimation. The action space of the microparts $U=\left\{v_{1},v_{2},v_{3},v_{4},v_{5}\right\}$ and the micropart cannot move to nodes that are occupied. The configurations of $A^{1}\;\&\;A^{2}$ microparts are equal to (1, 3) and (3, 3) respectively. The $A^{3}$ micropart starts from (2, 1) and its goal is to reach the node (5, 3). The algorithm selects the next node with the minimum value of the $f\left(q^{i^{\prime }},q^{w},\;t_{0}+s\Delta t\right)$ function. 

The algorithm examines all the feasible configurations and finally the $\;A^{3}$ micropart follows a route, which stands off considerably from the $\;A^{1}\;\&\;A^{2}$. The analytical computations of the algorithm are available in Table A1. That is included in Appendix A. In many cases the $g\left(q^{i^{\prime }}\right)$ function has the same value for more than one feasible nodes of the $\;A^{3}$. The $h\left(q^{i^{\prime }},q^{w},\;t_{0}+s\Delta t\right)$ heuristic function is the most significant for driving the $A^{3}$ micropart to nodes far from the nodes occupied by the $\;A^{1}\;\&\;A^{2}$. 

The $f\left(q^{i^{\prime }},q^{w},\;t_{0}+s\Delta t\right)$ function contributes to the selection of configurations, which are close to the goal and away from the rest microparts. This property contributes in the minimization of the time required so that the micropart to reach its goal. Specifically, the micropart between two configurations that have the same distance from the goal configuration will select the one which is in longer distance from the rest of moving microparts. This property will make it to stop fewer times in its current configuration, which optimizes the total time that is requested for the motion of the microparts. 

### 6.2. The Micro Cooperative A* Algorithm (μCA*)

The batch-parallel micromanipulation of multiple microparts aims at sorting and assembly MEMS applications. Specifically, during the process of MEMS synthesis the prioritizing of the handling of the individual microparts. which compose them is requested. Thus, a decoupled method is proposed for the parallel motion of multiple microparts on the “Smart Platform”. The micro-Cooperative-A* algorithm (μCA*) is a decoupled path finder that computes the route of the microparts in $\;X_{free}\;$ space by minimizing the total time of the parallel motion of the microparts. The microparts can be displaced discretely in the time-space and the goal of this method is to find paths for every micropart and to avoid as much as possible the microparts to remain stationary to their current configuration. 

A Pseudocode where the basic formulation of the μCA* algorithm is presented at the end of this Section in Algorithm 1. As it is shown in the Pseudocode the input to the algorithm is the graph, the Path space, which includes the path of the microparts with higher priority in time-space, the start and goal configuration of the considered micropart $\;m_{i}$, the start time $t_{0}$ and $t_{0}+\Delta t$ and the OPEN and CLOSE lists, which are empty at the beginning of the computations. In order to simplify the computations a quite large time-space is considered at so that T=S·Δt where S≫n.

The search is expanded in the specified time-space T and the algorithm computes the $\;Q^{i^{\prime }}$ space and then $\forall \;q_{t_{0}+s\Delta t\;}^{i}\in Q^{i^{\prime }}$ it is checked if $(q^{i\prime }\in \;q_{t_{0}+s\Delta t\;}^{i})\;\in “\mathrm{graph}”$ and $q_{t_{0}+s\Delta t\;}^{i}\notin$ CLOSE list. In the case, where both these conditions are satisfied, the algorithm keeps on its search. The algorithm recalls all the previous paths, which have already been specified for the microparts with higher priority, and their configurations at $t_{0}+\left(s-1\right)\Delta t$ and $\;t_{0}+s\Delta t$ are isolated. The constraints that are proposed in Table 3 are checked and if they are satisfied the algorithm goes on with the computation of the $f\left(q^{i^{\prime }},q^{w},\;t_{0}+s\Delta t\right)$ function. For the option that the configurations of the microparts do not accord with the constraints the algorithm skips the $\;q_{t_{0}+s\Delta t\;}^{i}$ configuration that is studied currently. For each configuration $q_{t_{0}+s\Delta t}^{i}$ that the function (12) is finally computed, the [$q_{t}^{i}$, $q_{t_{0}+s\Delta t}^{i}\;,f\left(q^{i^{\prime }},q^{w},\;t_{0}+s\Delta t\right)$] is temporally stored in the Temporary list and when the computations have been completed $\forall \;q_{t_{0}+s\Delta t}^{i}\in Q^{i^{\prime }}$ the content of the Temporary list with the minimum value of $f\left(q^{i^{\prime }},q^{w},\;t_{0}+s\Delta t\right)$ function is included in the OPEN list. At the end of these computations the $\;[q_{t}^{i}$, $q_{t_{0}+s\Delta t}^{i}\;,f\left(q^{i^{\prime }},q^{w},\;t_{0}+s\Delta t\right)$] content of the OPEN list is included in the CLOSE list so that not to be considered again by the algorithm. 

**Algorithm 1** Pseudocode for the μCA*CALL “graph” and **Specify**
k   {the total number of microparts} $t_{0}=\Delta t$; **Specify**
S i=1;
***While***
i≤k $w_{1}=i-1;$
  **If**
$w_{1}\neq 0$
   **For**
$w=1:w_{1}$  *Path* = **CALL**[*P*_1_, *P*_2_, .. *P_w_*] {The paths of the microparts in *T* with higher priority}   **End**  **End** $q^{i}=\;Start\_node$ of $m_{i};$, $q^{i,goal}=\;Goal\_node$ of $m_{i}$;  OPEN = [∅];,  CLOSE = [∅];
 **For**
s=1:S $Q^{i^{\prime }}\leftarrow \left\{\left(q^{i^{\prime }}=f\left(q^{i}\right)=q^{i}+v,\;\;\;v\;\in U\right),\;t_{0}+s\Delta t\;\right\}$  **For**
$q_{t_{0}+s\Delta t}^{i}\;\in \;Q^{i^{\prime }}$
   **If**
$\;\left(q^{i^{\prime }}\in \;q_{t_{0}+s\Delta t}^{i}\;\right)\in \mu \mathrm{graph}$ and ∉ CLOSE
    **if**
$w_{1}=0$
      $f\left(q^{i^{\prime }},t_{0}+s\Delta t\;\right)$
←
$\;\left(q^{i,goal}-\;q^{i\prime }\right)$
     **Else if**
$\;\;w_{1}\neq 0$      **For**
$w=1:w_{1}$     Find
$q_{t_{0}+\left(s-1\right)\Delta t}^{w}\;\mathrm{and}\;\;q_{t_{0}+s\Delta t}^{w}$
∈
Path       **If**
$q_{t_{0}+\left(s-1\right)\Delta t}^{i}$, $q_{t_{0}+\left(s-1\right)\Delta t\;}^{w},q_{t_{0}+s\Delta t}^{i}\;,q_{t_{0}+s\Delta t}^{w}\;\in$
Table 3        $f\left(q^{i^{\prime }},q^{w},t_{0}+s\Delta t\;\right)$
$\leftarrow \left(q^{i,goal}-\;q^{i\prime }\right)+\;\frac{i-1}{\sum_{w=1}^{i-1}\left|q_{t_{0}+s\Delta t}^{i}-q_{t_{0}+s\Delta t}^{w}\right|}$       **Else**        *Continue* {**Skip**$\;\;\;q_{t_{0}+s\Delta t}^{i}$}       **End**      **End**
     **End**    **If** [$q_{t_{0}+\left(s-1\right)\Delta t}^{i}$, $\;q_{t_{0}+s\Delta t}^{i}$,$\;f\left(q^{i},q^{i^{\prime }},q^{w},\;t_{0}+s\Delta t\right)$] ∈OPEN     *Continue* {Skip $\;\;q_{t_{0}+s\Delta t}^{i}$}    **End**
    Temporary list ← [$q_{t_{0}+\left(s-1\right)\Delta t}^{i}$, $q_{t_{0}+s\Delta t}^{i}$,$\;f\left(q^{i},q^{i^{\prime }},q^{w},\;t_{0}+s\Delta t\right)$];   **End**  **End**     **If**
Temporary list  = [∅]
      Print “**THERE IS NO PATH**”     **Else**
      For member
∈
Temporary list 
      Find “member” where $f\left(q^{i^{\prime }},q^{w},t_{0}+s\Delta t\right)=minimum$
      OPEN
← 
“member”      **End**     **End**    CLOSE
← 
“member”,  $q^{i}\leftarrow q^{i\prime }\;$($q^{i^{\prime }}\in \;\;q_{t_{0}+s\Delta t}^{i}\;\in$
“member”)   **End**
 $P_{i}=[\;q_{t_{0}}^{i},\left\{\;2^{nd}\mathrm{Column}\;\mathrm{elements}\;\mathrm{of}\;\mathrm{OPEN}\;\right\}],$ i++; **End**

## 7. Simulation Results—Discussion

In this Section the routes of multiple simultaneously moving microparts are computed with the μCA* using the proposed cost Equation (12). In the first example, three microparts are placed on a simulated 50 × 50 electrodes “Smart Platform” with 2 static obstacles on its surface. The start and goal configurations of the microparts are included in Table 4. The indicator of every micropart specifies its priority during the path computation. The Time Space is equal to $T=\left[t_{0},\;t_{0}+S\Delta t\right]$ and it is considered that S=100=2×50, thus T=[1, 101]. In order to simplify the computations, the time period Δt is equal to 1. 

The paths of the $m_{1}$, $m_{2}\;$ and $m_{3}$ are also computed with the μCA* using the conventional distance function of A* algorithm that is described by Equation (13) [23]. Considering the Pseudocode of the μCA* in Section 6, the algorithm does not expand to the cost computation, if the currently studying configuration is not a member of the Free State Space ($X_{free}$). Equation (13) is selected in order to be validated that even if the micropart moves in $X_{free}$, the distance between its current and goal configuration is not a sufficient criterion for estimating its next position. The computations of the μCA* for both Equations (12) and (13) are selected and compared. 

(13) $$f\left(q^{i},q^{i^{\prime }}\right)=\;\left(q^{i,start}-\;q^{i}\right)+\left(q^{i,goal}-\;q^{i\prime }\right).$$

**Figure 23:**
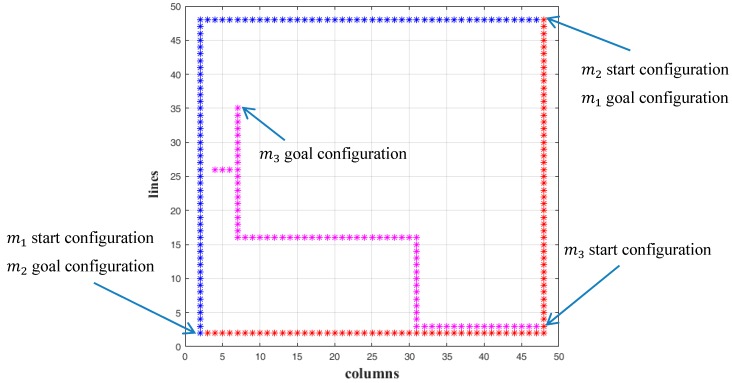
The 2D representation of the path computation of the three microparts with the computations of Equation (13).

**Figure 24:**
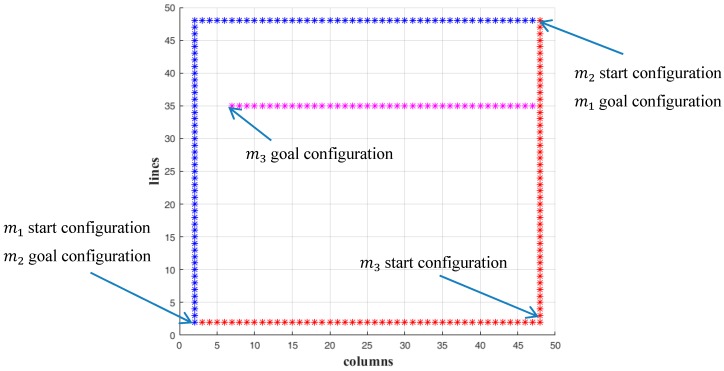
The 2D representation of the path computation of the three microparts with the computations of Equation (12).

**Figure 25:**
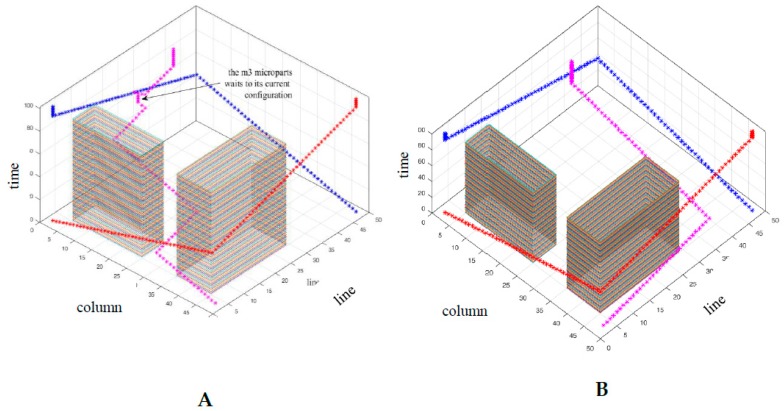
The path of the three microparts in T (time-space), (**A**) Equation (13) (**B**) Equation (12).

The results in 2D space are illustrated in Figure 23 and Figure 24 where the collision avoidance with the static obstacles (blue lines) is confirmed. In Figure 25A,B the path of the microparts is represented in time-space. As it is shown, the $m_{1}$, $m_{2}\;$ and $m_{3}$ reached their goal within  T and they were considered as static, since they reached their goal and until $\;t_{0}+100\Delta t=101$.

In both cases (Equations (12) and (13)—Figure 25A,B) the $m_{1}$ and $m_{2}\;$ moved with the same pattern and they completed their motion when $\;t_{0}+S\Delta t=1+92\cdot 1=93$ (the Manhattan metric is equal to: |48−2|+|48−2|=92), so they did not remain stable to any node of their route. On the other hand, the path of the $m_{3}$ in Figure 25A,B, is significantly different. At the beginning of its motion the Equation (13) drives the micropart to the left while the Equation (12) makes the $m_{3}\;$ to move up. As it is illustrated, the Equation (13) traps the $m_{3}\;$ to two configuration for seven and five times, steps respectively, while with the use of Equation (12) the $m_{3}\;$ succeeds to reach its goal without remaining stable anywhere. The results of the μCA* for the $\;m_{3}\;$ are included in Table 5. It is considerable that the Equation (12) succeeded to minimize the total time that is requested for the motion of $m_{3}$ while the Equation (13) did not provide the optimum results.

The μCA* does not expand its computations when there is no feasible configuration, which is member of the Free State Space to select for the micropart. The algorithm prints then that there is no route and the micropart is not considered anymore on the SP. The second example of this Section studies the contribution of the Equation (12) in the successive path computations of μCA* for multiple simultaneously moving microparts. Five microparts are placed on the 50 × 50 electrodes platform without static obstacles, S=100 and Δt=1. In Table 6 the input start and goal configurations of the microparts are included. The computations of μCA* with Equation (12) are compared with the corresponding computations of the algorithm with Equation (13). 

**Figure 26:**
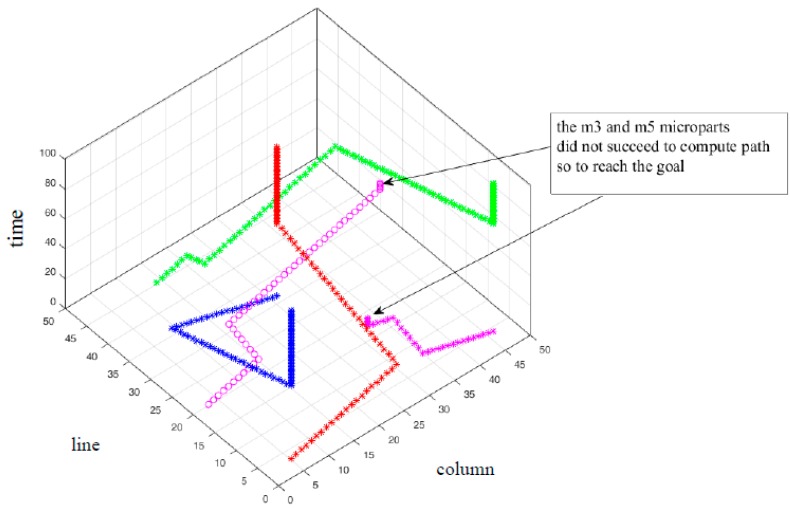
The path of the five microparts in T (time space) with the use of the Equation (13).

**Figure 27:**
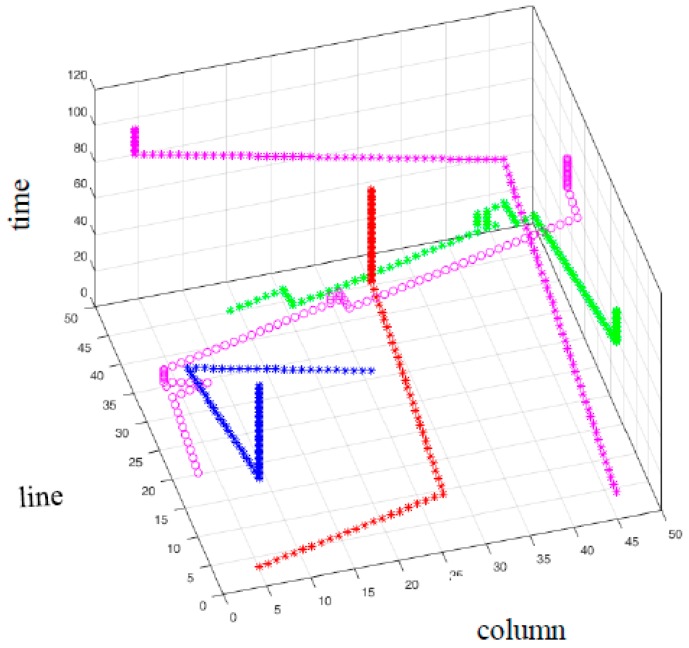
The path of the five microparts in T (time space) with the use of the Equation (12).

As it is illustrated in Figure 26 the Equation (13) did not contribute in the route determination of $m_{3}$ and $m_{5}$*—*the simulation results in Figure 26 illustrate the routes that were computed until the μCA* to print that “there is no path”. On the other hand, with the use of Equation (12) paths were determined for all the microparts (Figure 27). Comparing the graphs and the simulation results of Table 6, it is considerable that in the case of Equation (13), the $m_{4\;}\;$ completed its motion in optimum time. However, since the μCA* with Equation (13) did not provide path-solution for the $\;m_{3}$, the algorithm computed the path of the $m_{4\;}$ without taking into account the configurations of $\;m_{3}$, thus the μCA* expanded to $m_{4}$ route determination considering just the $m_{1}$ and $\;m_{2}$. Finally, in the case of $m_{5}\;$ the contribution of Equation (12) is significant, since the micropart succeeded to reach its destination avoiding being trapped by $m_{1}$,$\;m_{2}\;,\;m_{3}$ and $\;m_{4}$.

The results of the two simulated examples of this Section substantiate that the Equation (13) may contribute in optimum computations of the μCA* when the complexity of the problem is low (two microparts). So, it is verified that cost functions similar to Equation (13) are insufficient for the path computation of multiple simultaneously moving microparts on the SP. In the case of Equation (12) it is validated that the identity of the new heuristic function to “predict” the free area of the SP, makes the microparts to move in parallel with secure, following safe optimum routes and finally reach their goal configuration. 

## 8. Conclusions

An approach is introduced for the micromanipulation of multiple microparts on a “Smart Platform” where circular conductive electrodes are embedded that are activated to drag the microparts to the next positions. We proposed a new layout of the electrodes underneath the microparts, which decreased the mass of the microparts. Regarding our previous work a more accurate study about the physical parameters of the SP is presented and the magnitude of the applied potential, which makes the microparts to leave their static condition considering their dimensions is determined. According to the new layout, the activation algorithms for the up, down, left and right motion of the microparts on the platform, is presented. 

The Configuration-Space (C-Space) of the microparts on the platform considering their dimensions and the Static-Obstacles on the SP was computed. Taking into account the C-Space the graph of the “Smart Platform” was specified. Moreover, the constraints for the configurations of the in parallel moving microparts are proposed and their Free State Space on the SP is determined. Considering the Free-State-Space a modified Cooperative A* algorithm (μCA*) is proposed. The innovation of the introduced route finding method is a new heuristic function, which considers both the Manhattan metric from the feasible position to the goal configuration and to the rest moving microparts in time-space. The μCA* path search is implemented applying both the new heuristic function and the conventional distance function of the A* algorithm.

## 9. Discussion—Future Work

This work proposes a complete method for the parallel motion of multiple rectangular microparts with electrostatic fields on the “Smart Platform”. The path planning and motion planning of the microparts considering all the constraints, which are presented due to the activation method of the SP electrodes is described for the first time. The Simulation Results that are presented in Section 6 demonstrate that the new heuristic function succeeds to minimize the time when the microparts reach their goal configuration and to find route for multiple microparts (compared to the conventional distance function of the A* algorithm).

Considering the publications that were discussed in the introduction of this work, it is substantiated that the parallel manipulation of multiple microparts exclusively with electrostatic fields on an electrodes array has not been practically validated yet. Thus, it is a great task for our team to construct the “Smart Platform”, taking into account the design that we have proposed in our publications and the determinations that have resulted from F.E.M. analysis. Moreover, future work will be concentrated on manipulation methods for the batch parallel sorting and assembly applications. The microparts will be moved to the desired regions of the SP with the μCA* algorithm and then with an automated activation of the SP electrodes will be handled either to be sorted or assembled. 

## Figures and Tables

**Table 1:** Finite Elements Method (F.E.M.) measurements and computations of the Forces applied to rectangular microparts of different sizes (the three significant digits are considered).

*r* (μm)	Potential V+ (Volts)	$F_{elec,vertical}^{2}\left(d_{e}\right)\;(\mu N)$	Micropart’s Weight (μN)	Static Friction Force T (μN)	$F_{elec}^{2}\;\left(\begin{array}{c} d_{e}=\\ 2r+d_{1}r \end{array}\right)\;(\mu N)$	$F_{elec}^{2}\;\left(d_{e}\right)-T\;(\mu N)$
15	50	0.408	0.00874	0.125	0.55	0.425
20	50	0.424	0.0207	0.134	0.451	0.318
25	100	0.404	0.0405	0.1335	0.646	0.513
30	100	0.409	0.0699	0.144	0.65	0.508
35	100	0.406	0.111	0.155	0.747	0.592
40	100	0.414	0.166	0.174	0.747	0.573
45	100	0.418	0.236	0.196	0.743	0.546
50	125	0.694	0.324	0.365	1.072	0.707
55	125	0.882	0.431	0.394	1.065	0.665
60	150	0.93	0.559	0.447	1.475	1.0285
65	150	0.905	0.711	0.485	1.482	0.998
70	175	1.606	0.888	0.748	1.797	1.042
75	175	1.611	1.092	0.811	1.952	1.141
80	175	1.599	1.326	0.878	1.973	1.096
85	175	1.802	1.589	1.258	2.193	0.935
90	200	2.512	1.887	1.312	3.154	1.842
95	200	2.552	2.212	1.431	3.192	1.761
100	225	2.508	2.589	1.529	3.817	2.288

**Table 2:** Activation Methods of the “Smart Platform” (SP) electrodes for the right/left and down/up motion of the microparts.

	Next Configuration $\left(q^{i}{}^{\prime }\right)$	Activated Electrodes
$\mathrm{Current}\;\mathrm{Configuration}\;q^{i}\;=\left(c^{i},l^{i}\right)$	$\;q^{i}+v_{1}$ (right motion)	$\;\left(c^{i}+2,l^{i}-1\right)\;\left(c^{i}+2,l^{i}+1\right)$ (Figure 11A)
	$\;q^{i}+v_{2}$ (left motion)	$\;\left(c^{i}-2,l^{i}-1\right)\left(c^{i}-2,l^{i}+1\right)$ (Figure 11B)
	$\;q^{i}+v_{3}$ (down motion)	$\;\left(c^{i}-1,l^{i}-2\right)\left(c^{i}+1,l^{i}-2\right)$ (Figure 11C)
	$\;q^{i}+v_{4}$ (up motion)	$\;\left(c^{i}-1,l^{i}+2\right)\left(c^{i}+1,l^{i}+2\right)$ (Figure 11D)
	$\;q^{i}+v_{5}$ (remain stable)	“No SP Electrodes are Activated”

**Table 3:** Possible scenarios for the collision avoidance between the two simultaneously moving microparts.

**Current Configuration** $q_{t_{0}+\left(s-1\right)\Delta t}^{i}=\left(c^{i},\;l^{i},t_{0}+\;\left(s-1\right)\Delta t\right)=\left(q^{i},\left(s-1\right)\Delta t\right)$ **of the** $m_{i}$ **Micropart and the Current Configuration of the** $m_{j}\;$ $\;\;q_{t_{0}+\left(s-1\right)\Delta t}^{j}=\left(c^{j},\;l^{j},t_{0}+\left(s-1\right)\Delta t\right)$ **=** $\left(q^{j},\left(s-1\right)\Delta t\right)$	**Next Configuration**	**Constraints to the Next Configuration**
	$q_{t_{0}+s\Delta t}^{i}=\left(c^{i},\;l^{i},t_{0}+s\Delta t\right)$ of the $\;\;\;m_{i}\;$ Micropart	$\;\;q_{t_{0}+\left(s-1\right)\Delta t}^{j}=\left(c^{j},\;l^{j},t_{0}+s\Delta t\right)$ of the $\;\;\;m_{j}\;$ Micropart
	$\;q^{i}+v_{1}$	$q^{j}+v_{1\;}$ when $\;c^{i}-c^{j}\geq 5$, $q^{j}+v_{2}$ when $\;\;c^{i}-c^{j}\geq 6,$ $q^{j}+v_{3}\;$ when $c^{i}-c^{j}\geq 6\;or\;\;\;l^{i}-l^{j}\geq 6$ $q^{j}+v_{4}\;$ when $c^{i}-c^{j}\geq 4\;or\;\;\;l^{i}-l^{j}\geq 4\;$ $q^{j}+v_{5}\;$ when $\;c^{i}-c^{j}\geq 4$
	$\;q^{i}+v_{2}$	$q^{j}+v_{1\;}$ when $\;c^{i}-c^{j}\geq 6$, $q^{j}+v_{2}$ when $\;\;c^{i}-c^{j}\geq 5,$ $q^{j}+v_{3}\;$ when $c^{i}-c^{j}\geq 4\;\;\;or\;\;\;\;l^{i}-l^{j}\geq 4$ $q^{j}+v_{4}\;$ when $c^{i}-c^{j}\geq 6\;\;or\;\;\;\;l^{i}-l^{j}\geq 6$ $q^{j}+v_{5}\;$ when $\;c^{i}-c^{j}\geq 5$
	$\;q^{i}+v_{3}$	$q^{j}+v_{1\;}$ when $c^{i}-c^{j}\geq 4\;or\;\;\;\;l^{i}-l^{j}\geq 4$ $\;q^{j}+v_{2}\;$ when $c^{i}-c^{j}\geq \;6\;or\;\;\;\;l^{i}-l^{j}\geq 6$ $q^{j}+v_{3}\;$ when $\;\;l^{i}-l^{j}\geq 5,\;\;q^{j}+v_{4}\;$ when $\;\;l^{i}-l^{j}\geq 6$ $q^{j}+v_{5}\;$ when $\;l^{i}-l^{j}\geq 5$
	$\;q^{i}+v_{4}$	$q^{j}+v_{1\;}$ when $c^{i}-c^{j}\geq 6\;or\;\;\;\;l^{i}-l^{j}\geq 6$ $\;q^{j}+v_{2}\;$ when $c^{i}-c^{j}\geq 4\;or\;\;\;\;l^{i}-l^{j}\geq 4$ $q^{j}+v_{3}\;$ when $\;\;l^{i}-l^{j}\geq 6$, $\;q^{j}+v_{4}\;$ when $\;\;l^{i}-l^{j}\geq 5\;$ $q^{j}+v_{5}\;$ when $\;l^{i}-l^{j}\geq 4$
	$\;q^{i}+v_{5}$	$q^{j}+v_{1\;}$ when $c^{i}-c^{j}\geq 5$, $\;q^{j}+v_{2}$ when $\;\;c^{i}-c^{j}\geq 4,$ $q^{j}+v_{3}\;$ when $\;\;l^{i}-l^{j}\geq 4$, $\;q^{j}+v_{4}\;$ when $\;\;l^{i}-l^{j}\geq 5$ $q^{j}+v_{5}\;$ when $\;c^{i}-c^{j}\geq 5\;or\;\;\;l^{i}-l^{j}\geq 5\;$

**Table 4:** Start and Goal Configurations of the 4 Moving Microparts.

Micropart Number—Symbol Figure	Start Configuration $\mathrm{When}\;t_{0}=1$ $\left(q_{1\;}^{i}\forall \;i\in \left[1,3\right],i\in \mathbb{N}\right)$	Goal Configuration $q^{i}\forall \;i\in \left[1,3\right],i\in \mathbb{N})$
Micropart 1 (red * its COM in Figure 23)	$q_{1}^{1}=$ (2,2,1)	$q_{101}^{1}=$ (48,48,101)
Micropart 2 (blue * its COM in Figure 23)	$q_{1}^{2}=$ (48,48,1)	$\;q_{101}^{2}=2\left(2,2,101\right)$
Micropart 3 (pink * its COM in Figure 23)	$q_{1}^{3}=$ (48,3,1)	$\;q_{101}^{3}=\left(7,35,101\right)$

**Table 5:** Start and goal configurations of the third micropart, comparing functions (11) and (12).

Micropart	Goal Configuration after Path Computation with (13)	Goal Configuration after Path Computation with (12)	Difference of Time Steps
$\;m_{3}$	$\;q_{86}^{3}=\left(7,35,86\right)$	$q_{74}^{3}=$ (7,35,74)	86−74=12

**Table 6:** Start, goal configurations, Manhattan metric and results of the five moving microparts.

$m_{i\;},\;i=\left[1,5\right].$ (Symbol)	$\;q_{1}^{i}\;$	$\;q_{101}^{i}\;$	Manhattan Metric	$q_{end}^{i}\;\mathrm{Equation}\;(13)$	$q_{end}^{i}\;\mathrm{Equation}\;(12)$
$m_{1\;}$(red *)	(5,3,1)	(26,31,101)	49	(26,31,50)	(26,31,50)
$m_{2}$ (blue*)	(26,31,1)	(5,3,101)	49	(5,3,50)	(5,3,50)
$m_{3\;}$(pink *)	(46,4,1)	(4,48,101)	86	(27,11,35)	(46,4,88)
$m_{4\;}$(green *)	(14,45,1)	(46,4,101)	73	(46,4,74)	(46,4,86)
$m_{5\;}$(pink ^o^)	(3,20,1)	(48,30,101)	55	(43,27,74)	(48,30,86)

* COM of $m_{1\;}m_{2\;}m_{3\;\;}m_{4\;}$. ° COM of $m_{5\;}$.

## Appendix A

**Table A1:** Analytical Computations with the new heuristic function.

$A^{3}$ Current	Feasible	$g\left(q^{i^{\prime }}\right)$ Considering $q^{goal}=\;(3,5)$	$h\left(q^{i^{\prime }},q^{w},\;t^{\prime }\right)$ Considering the Configurations of $A^{1}\;\&\;A^{2}$ Agents	$\;f\left(q^{i^{\prime }},q^{w},\;t^{\prime }\right)\;$
(2,1)	(1,1)	5	$\;\frac{2}{6}=\;\frac{1}{3}$	5.333
	(2,2)	4	$\;\frac{2}{4}=\;\frac{1}{2}$	4.5
	(2,1)	5	$\;\frac{2}{6}=\;\frac{1}{3}$	5.333
	(3,1)	4	$\;\frac{2}{6}=\;\frac{1}{3}$	4.333
(3,1)	(3,2)	3	$\;\frac{2}{5}$	3.4
	(4,1)	3	$\;\frac{2}{6}=\;\frac{1}{3}$	3.333
	(3,1)	4	$\;\frac{2}{6}=\;\frac{1}{3}$	4.333
	(2, 1)	5	$\;\frac{2}{6}=\;\frac{1}{3}$	5.333
(4,1)	(5,1)	2	$\;\frac{2}{10}=\;\frac{1}{5}$	2.2
	(3,1)	4	$\;\frac{2}{6}=\;\frac{1}{3}$	4.333
	(4,1)	3	$\;\frac{2}{6}=\;\frac{1}{3}$	3.333
	(4,2)	2	$\;\frac{2}{5}$	2.4
(5,1)	(4,1)	3	$\;\frac{2}{6}=\;\frac{1}{3}$	3.333
	(5,1)	2	$\;\frac{2}{10}=\;\frac{1}{5}$	2.2
	(5,2)	1	$\;\frac{2}{8}=\;\frac{1}{4}$	1.25
(5,2)	(4,2)	2	$\;\frac{2}{5}$	2.4
	(5,2)	1	$\;\frac{2}{8}=\;\frac{1}{4}$	1.25
	(5,3)	0	$\;\frac{2}{6}=\;\frac{1}{3}$	0.333
	(5,1)	2	$\;\frac{2}{10}=\;\frac{1}{5}$	2.2
